# Supplementary material for: SPECT/CT Radiomics for Differentiating between Enchondroma and Grade I Chondrosarcoma
Source: Tomography. 2023 Oct 16;9(5):1868–75. doi: 10.3390/tomography9050148 (PMC10610631; doi:10.3390/tomography9050148)
Supplement: Supplementary file 1 [file tomography-09-00148-s001.zip › ESM 1.pdf]

ESM Table 1. List of extracted metabolic parameters

Conventional SUV parameters	SUVmax<br>SUVmean<br>Coefficient of Variation<br>TLU<br>Volume
Histology	Skewness<br>Kurtosis<br>Entropy<br>Energy<br>Sphericity<br>Compacity
Gray-Level Co-occurrence Matrix (GLCM)	Homogeneity<br>Energy<br>Contrast<br>Correlation<br>Entropy<br>Dissimilarity
Grey-Level Run Length Matrix (GLRLM)	Short Run Emphasis<br>Long Run Emphasis<br>Low Gray-level Run Emphasis<br>High Gray-level Run Emphasis<br>Short-Run Low Gray-level Emphasis<br>Short-Run High Gray-level Emphasis<br>Long-Run Low Gray-level Emphasis<br>Long-Run High Gray-level Emphasis<br>Gray-Level Non-Uniformity<br>Run Length Non-Uniformity<br>Run Percentage
Neighborhood Grey-Level Different Matrix (NGLDM)	Coarseness<br>Contrast<br>Busyness
Grey-Level Zone Length Matrix (GLZLM)	Short-Zone Emphasis<br>Long-Zone Emphasis<br>Low Gray-level Zone Emphasis<br>High Gray-level Zone Emphasis<br>Short-Zone Low Gray-level Emphasis<br>Short-Zone High Gray-level Emphasis<br>Long-Zone Low Gray-level Emphasis<br>Long-Zone High Gray-level Emphasis<br>Gray-Level Non-Uniformity<br>Zone Length Non-Uniformity<br>Zone Percentage
